# Immune Monitoring of the Circulation and the Tumor Microenvironment in Patients with Regionally Advanced Melanoma Receiving Neoadjuvant Ipilimumab

**DOI:** 10.1371/journal.pone.0087705

**Published:** 2014-02-03

**Authors:** Ahmad A. Tarhini, Howard Edington, Lisa H. Butterfield, Yan Lin, Yongli Shuai, Hussein Tawbi, Cindy Sander, Yan Yin, Matthew Holtzman, Jonas Johnson, Uma N. M. Rao, John M. Kirkwood

**Affiliations:** 1 Department of Medicine, University of Pittsburgh Cancer Institute, Pittsburgh, Pennsylvania, United States of America; 2 Department of Surgery, West Penn Allegheny Health System, Pittsburgh, Pennsylvania, United States of America; 3 Department of Biostatistics, University of Pittsburgh Graduate School of Public Health, Pittsburgh, Pennsylvania, United States of America; 4 Department of Surgery, University of Pittsburgh Medical Center, Pittsburgh, Pennsylvania, United States of America; 5 Department of Otolaryngology, University of Pittsburgh School of Medicine, Pittsburgh, Pennsylvania, United States of America; 6 Department of Pathology, University of Pittsburgh School of Medicine, Pittsburgh, Pennsylvania, United States of America; Université Paris Descartes, France

## Abstract

We evaluated neoadjuvant ipilimumab in patients with surgically operable regionally advanced melanoma in order to define markers of activity in the blood and tumor as assessed at baseline (before ipilimumab) and early on-treatment. Patients were treated with ipilimumab (10 mg/kg intravenously every 3 weeks ×2 doses) bracketing surgery. Tumor and blood biospecimens were obtained at baseline and at surgery. Flow cytometry and immunohistochemistry for select biomarkers were performed. Thirty five patients were enrolled; IIIB (3; N2b), IIIC (32; N2c, N3), IV (2). Worst toxicities included Grade 3 diarrhea/colitis (5; 14%), hepatitis (2; 6%), rash (1; 3%), elevated lipase (3; 9%). Median follow up was 18 months: among 33 evaluable patients, median progression free survival (PFS) was 11 months, 95% CI (6.2–19.2). There was a significant decrease in circulating myeloid derived suppressor cells (MDSC). Greater decrease in circulating monocyte gate MDSC Lin1−/HLA-DR−/CD33+/CD11b+ was associated with improved PFS (p = 0.03). There was a significant increase in circulating regulatory T cells (Treg; CD4+CD25hi+Foxp3+) that, unexpectedly, was associated with improved PFS (HR = 0.57; p = 0.034). Baseline evidence of fully activated type I CD4^+^ and CD8^+^ antigen-specific T cell immunity against cancer-testis (NY-ESO-1) and melanocytic lineage (MART-1, gp100) antigens was detected and was significantly potentiated after ipilimumab. In tumor, there was a significant increase in CD8^+^ T cells after ipilimumab (p = 0.02). Ipilimumab induced increased tumor infiltration by fully activated (CD69^+^) CD3^+^/CD4^+^ and CD3^+^/CD8^+^ T cells with evidence of induction/potentiation of memory T cells (CD45RO^+^). The change in Treg observed within the tumor showed an inverse relationship with clinical benefit and greater decrease in tumor MDSC subset Lin1−/HLA-DR−/CD33^+^/CD11b^+^ was associated with improved PFS at one year. Neoadjuvant evaluation revealed a significant immunomodulating role for ipilimumab on Treg, MDSC and effector T cells in the circulation and tumor microenvironment that warrants further pursuit in the quest for optimizing melanoma immunotherapy.

## Introduction

Patients with palpable regional lymphatic involvement with melanoma (AJCC stage IIIB-C) carry a risk of relapse and death that approaches 70% at 5 years [1]. The development of local or regional recurrence after initial surgical management portends an even poorer prognosis [2]–[4]. In the Melanoma Surgical Trial, a local recurrence was associated with 5 and 10 year survival rates of 9–11% and 5%, respectively [3].

Neoadjuvant therapy has been demonstrated to improve outcome in the management of patients with multiple different solid tumors [5]–[8]. A further significant advantage of the neoadjuvant approach in relation to translational investigations of new agents is the ability to evaluate the clinical and pathologic responses, and the access to tumor tissue before and after neoadjuvant therapy. This allows a direct assessment of the antitumor mechanisms that may enable selective application of therapeutic agents to those patients most likely to benefit.

Ipilimumab is a human immunoglobulin-G (IgG1)κ anti-CTLA-4 monoclonal antibody. It was approved by the U.S. FDA for the treatment of advanced inoperable melanoma in March 2011 at a dose of 3 mg/kg given every 3 weeks for 4 doses, based on the results of a phase III trial [9]. Prior data suggested a dose dependent effect of ipilimumab from 0.3 mg/kg to 10 mg/kg, where 10 mg/kg appeared to have the greatest efficacy, but the rate of high-grade immune related adverse events (irAEs) was also dose dependent [10]. Based on these and other data, ipilimumab at 10 mg/kg was taken forward for phase II and phase III studies including the CA184024 trial in metastatic disease combined with dacarbazine [11] and the adjuvant trials EORTC18071 and E1609 [12], [13].

We had previously reported a significant immunomodulatory impact of a CTLA4 blockade-based regimen on circulating myeloid-derived suppressor cells (MDSC) and regulatory T cells (Treg) in metastatic melanoma patients treated with that regimen unlike patients treated with a peptide vaccination-based regimen [14]. We hypothesized that similar changes in MDSC and Treg may be observed with ipilimumab in the neoadjuvant/adjuvant setting that may be best evaluated in a neoadjuvant study that provides parallel evaluation in the circulation and the tumor microenvironment (TME) of suppressor and effector immune cells.

We have therefore conducted a neoadjuvant translational evaluation of ipilimumab at 10 mg/kg in patients with locally and/or regionally advanced melanoma, with the primary goal of generating biomarker data and providing a preliminary assessment of efficacy and safety of neoadjuvant ipilimumab as secondary endpoints. Primarily, we pursued the monitoring of cellular markers of immunosuppression and of effector T cells before and after ipilimumab as assessed in the TME and in the circulation, testing the hypothesis that these biomarkers will be significantly modulated and may have therapeutic predictive roles. Such findings may be further tested in larger adjuvant trials involving ipilimumab.

## Patients and Methods

### Patients

#### Ethics statement

The study was approved by the University of Pittsburgh Institutional Review Board (IRB; IRB# PRO09010033). All patients had a University of Pittsburgh IRB approved written informed consent obtained. The study was conducted in accordance with the principles expressed in the Declaration of Helsinki. Eligible patients were 18 years or older and had clinically detectable locally and/or regionally advanced melanoma (cutaneous, mucosal or unknown primary).

### Study Design and Treatment

A pre-treatment biopsy was required followed by 2 doses of induction therapy with ipilimumab at 10 mg/kg intravenously administered 3 weeks apart. Surgery was planned between weeks 6–8 following initiation of ipilimumab. Two doses of maintenance ipilimumab were planned to be given 3 weeks apart following recovery from surgery. Adjuvant high dose interferon-α (HDI) was allowed in interferon-α naïve patients. Blood specimens for this project were collected at baseline and 6 weeks. Blood was drawn into heparin (for peripheral blood mononuclear cells; PBMC) tubes or tubes without anticoagulant (serum) and processed by the Immunologic Monitoring Lab upon receipt. Tumors were transported to the Tissue Procurement Facility after surgery in sterile medium. Part of the tumor was formalin-fixed and paraffin-embedded for immunohistochemistry, and part was enzymatically digested to single cells and cryopreserved for Treg, MDSC and T cell analysis.

### Toxicity and Response Assessments

The descriptions and grading scales found in the NCI Common Terminology Criteria for Adverse Events version 3.0 were utilized for AE grading and reporting. For the purpose of response assessment (modified WHO criteria), imaging studies were carried out at baseline (before ipilimumab), 6–8 weeks after starting ipilimumab (before surgery) then at 3 month intervals. Responses were not confirmed radiologically due to the planned surgery. Responses were classified as complete response (CR), partial response (PR), stable disease (SD) or disease progression (PD). Clinical benefit was defined as CR, PR or SD.

### Statistical Methods

The study aimed to assess the effects of ipilimumab on the host immune response, comparing assessments in the patients' peripheral blood and tumor at baseline with corresponding follow up evaluations made after 6 weeks of starting ipilimumab. The study was planned for at least 28 patients with the ability to replace some patients without adequate baseline tumor biopsy. A study of 28 patients was estimated to provide a 90% power to detect (at significance level 0.05) a 1.63 fold to 1.78 fold increase in average cell counts after 6 weeks of starting ipilimumab. The corresponding detectable reductions in cell counts would range from 0.56 to 0.61.

Baseline descriptive statistics on all evaluable patients were provided on demographic variables, laboratory parameters, toxicity and efficacy. Survival and recurrence-free survival were estimated by the Kaplan-Meier method.

### Laboratory Methods and Corresponding Statistical Analyses

*Multicolor flow cytometry* was used to compare cellular marker expression on thawed PBMC and infiltrated tumor, before and after treatment, focusing on circulating and intra-tumor regulatory T cells (Treg) and myeloid derived suppressor cells (MDSC). Healthy donor controls were run with the patient assays, according to laboratory SOPs. Treg were defined as lymphocytes expressing (1) CD4^+^/CD25hi^+^/intracellular FOXP3^+^ or (2) CD4^+^/CD25hi^+^/CD39^+^ [15] compared to activated T cells which expressed (CD3^+^CD4^+^CD25^+^). For the purposes of this study, MDSC were defined as cells expressing (1) Lin1^−^/HLA-DR^−^/CD33^+^/CD11b^+^ lymphoid (small FSCxSSC) gate [16], (2) Lin1^−^/HLA-DR^−^/CD33^+^/CD11b^+^ monocyte (larger FSCxSSC) gate [17]–[19] or (3) HLA-DR^+^^lo^/CD14^+^ monocyte gate [20]–[24]. In a subset of patients with sufficient cells, TIL were tested for activation, memory, cytokine or effector molecule expression (CD3, CD4, CD8, CD69, CD45RO, IFNγ, TNFα, CD107a, granzyme-B, perforin). Daily FC500-flow cytometer QC was run using Beckman-Coulter Flow-Check, Flow-Check675 and Flow-Check770 for laser alignment verification. Beckman-Coulter Flow-Set fluorospheres were used to standardize voltages to ensure consistency from day-to-day. Single-stained Beckman-Coulter Immuno-Trol control cells were used to establish compensation settings. For Treg analysis from thawed PBMC, cells were surface stained for CD4 and CD25 with or without CD39 (Beckman-Coulter), then permeabilized and stained for intracellular FOXP3 according to manufacturer's instructions (eBioscience Foxp3 Staining Buffer-Set). The lymphocytes were gated by FSCxSSC, then CD4^+^ cells were gated, then assessed for CD25-high and FOXP3 positivity (with dot-plot and histogram gates set using isotype control antibody stains). In addition, surface CD39 was also tested. The “% circulating Treg” defined here was the % of total CD4+ lymphocytes which were also CD25-high and FOXP3 positive. For MDSC analysis from thawed PBMC, cells were surface-stained for A) lineage cocktail (Becton-Dickinson, CD3/CD14/CD16/CD19/CD20/CD56), CD11b, HLA-DR (both Beckman-Coulter) and CD33 (Becton-Dickinson); or B) CD14 (Beckman-Coulter) and HLA-DR. Cells from tube-“A” were then gated by FSCxSSC for either lymphocytes (“lymphoid gate MDSC”) or myeloid cells (“monocyte gate MDSC”), then for lineage-negative+HLA-DR-negative, and then the myeloid subset assessed for CD11b^+^/CD33^+^ cells (with dot-plot and histogram gates set by isotype control antibody stains). Cells from “B” were then gated on by FSCxSSC for myeloid cells, then for CD14^+^/HLA-DR^lo^ cells (with dot-plot and histogram gates set by isotype control antibody stains). We used Beckman- Coulter CXP Software version 2.1 and Beckman-Coulter Kaluza Software version 1.2. Our goal for the cells of interest was 20,000 gated events with a minimum of 10,000 gated events. Examples of raw data are provided in Figure S3 (T-reg gating), Figure S4 (MDSC gating) and Figure S5 (Antigen-specific CD4^+^ and CD8^+^ T cell gating) (online only). Within-patient changes in Treg and MDSC from baseline to week-6 were tested by Wilcoxon signed-rank test. Within-patient changes in Treg and MDSC were also compared between the patients with clinical benefit (CR/PR/SD) and those with progression using the two-sample Wilcoxon rank-sum test. Statistical analyses were performed using SAS (SAS Institute Inc., Cary, NC). A significance level was set at 0.05 and all P-values reported were 2-sided and unadjusted.

To measure CD4^+^ and CD8^+^ T cells specific to tumor antigens (Gp-100, MART-1, NY-ESO-1) in an HLA-unrestricted fashion, libraries of 15-mer peptides overlapping by 4 amino acids were constructed (Mimetopes, Minneapolis, MN), after Palucka et al [25], [26]. PBMC from baseline and 6 weeks, or infiltrated tumor cells were co-cultured with peptide pools for 4 hours with monensin, and then stained for surface CD3, CD4, CD8 (Beckman Coulter), CD69 (BD) and intracellular IFNγ (eBiosciences). Controls included unstimulated cells and PMA/ionomycin-stimulated PBMC. Within-patient changes were tested by Wilcoxon signed-rank tests.

#### Immunohistochemistry

Paraffin-embedded tissue sections (5 µm thick) were scored at 20× magnification by a surgical pathologist (U.R.) who was blinded to patient and treatment status. Immunohistochemical stains were performed with mouse (FOXP3, Abcam Cat#ab22510; CD4, Dako Cat#M7310; CD8, Dako Cat#M7103; CD11c, Leica Cat#NCL-L-CD11C-563; CD45RO, Abcam Cat#ab23; CD20, Dako Cat#M075529-2) and rabbit (CD86, Epitomics Cat#1858-1) monoclonal antibodies as previously published [27]. The total numbers of intratumoral, peritumoral, and perivascular mononuclear cell infiltrates were enumerated at 20× magnification and actual numbers determined in four quadrants of the tissue, which often encompassed the entire tissue section. All internal and external controls were appropriate. Intensity of stains was optimal. The Wilcoxon singed-rank test was used to compare the level of each marker between week 6 and baseline. The Wilcoxon rank-sum test was used to compare the marker level between patients with clinical benefit and those with progression.

## Results

### Patient Characteristics

Thirty-five patients were enrolled between 2/2010 and 10/2012. Eight patients were newly diagnosed and 27 had recurred after prior treatment for melanoma that included surgery. One patient had unknown primary melanoma, 5 mucosal and 29 had cutaneous primaries including 3 with acral lentiginous melanoma. Eighteen patients had a component of in-transit metastatic melanoma. Overall staging/classification at study entry for those newly diagnosed or as an estimated risk classification for those with recurrent disease was IIIB (3; N2b) or IIIC (32; N2c, N3). For two patients, retrospective review demonstrated stage IV disease (Table 1).

**Table 1:** Patient Demographics and Baseline Disease Characteristics (N = 35 patients).

Variable	No. of Patients (%)
Age, years; Median (range)	52 (30–87)
Cutaneous primary	29 (83)
Mucosal primary	5 (14)
Unknown primary	1 (3)
Gender: Female	13 (37)
Gender: Male	22 (63)
Performance status (ECOG): 0	22 (63)
Performance status (ECOG): 1	13 (37)
Recurrent disease after prior surgery	27 (77)
Prior adjuvant HDI*	12 (34)
Presence of in-transit metastases	18 (51)
Estimated risk stage: IIIB	3 (9)
Estimated risk stage: IIIC	30 (86)
Estimated risk stage: IV (non-evaluable for efficacy)	2 (5)
Tumor Mutational Status: BRAF^V600^	15 (43)
Tumor Mutational Status: NRAS^Q61^	9 (26)
Tumor Mutational Status: NRAS ^R73^	1 (3)
Tumor Mutational Status: Unknown	3 (9)

* HDI: high dose interferon-α; ECOG: Eastern Cooperative Oncology Group;

### Treatment Details

A total of 106 cycles of ipilimumab were administered (median 4/patient) (Table S1).

### Efficacy

Two patients with stage IV disease were considered non-evaluable for efficacy according to study protocol criteria, including one cutaneous melanoma patient with bone marrow metastatic involvement and one patient with uterine cervical mucosal melanoma with pulmonary metastases. For the remaining 33 patients, the preoperative radiologic assessment (mWHO; unconfirmed) by PET-CT scans 6–8 weeks after the initiation of ipilimumab revealed 3 patients (9%) with an objective response (2 CR and 1 PR). Twenty-one patients (64%) had stable disease and eight patients (24%) had disease progression. All patients had histologically documented residual melanoma at definitive surgery following 2 doses of ipilimumab, although 5 patients had only microscopic disease detected. The median follow-up for patients at risk of progression was 17.6 months and for patients who were still alive was 16.1 months. The median progression-free survival (PFS) was 10.8 months, 95% CI (6.2, 19.2). The probability of 6 and 12 months PFS was 0.72, 95% CI (0.53, 0.84) and 0.47, 95% CI (0.29, 0.63). The probability of overall survival (OS) at 6 and 12 months was 0.97, 95% CI (0.78, 0.99). Figure S1 shows the Kaplan–Meier plot of the probability of PFS. Six patients who developed disease progression during follow up on study achieved objective response or NED status with subsequent therapy (Table S2).

### Safety

Table 2 summarizes by severity the adverse events (AEs) that were considered related to ipilimumab. No grade 4 or 5 immune related AEs were observed. Autoimmune toxicities were successfully managed with corticosteroids.

**Table 2:** Adverse events (worst grade) that were possibly, probably or definitely related to ipilimumab (N = 35; incidence ≥2)*.

Type	Sub-type	All Grades		Grade 1		Grade 2		Grade 3	
		No. Pts.	%	No. Pts.	%	No. Pts.	%	No. Pts.	%
**Immune mediated**	Adrenal Insufficiency	2	6	0	0	2	6	0	0
	Amylase/Lipase	5	14	1	3	1	3	3	9
	Diarrhea/Colitis	20	57	9	26	6	17	5	14
	Hepatitis AST/ALT/AP/GGT	3	10	0	0	1	3	2	6
	Hyper/pothyroidism	2	6	1	3	1	3	0	0
	Hypopituitarism (low ACTH), Hypophysitis	2	6	0	0	2	6	0	0
	Skin rash	20	57	11	31	8	23	1	3
**Constitutional**	Fatigue	12	34	5	14	6	17	1	3
	Fever	6	17	4	11	2	6	0	0
	Pruritus	18	51	13	37	5	14	0	0
**Other Gastrointestinal**	Nausea	8	23	7	20	0	0	1	3
	Vomiting	4	11	2	6	1	3	1	3
**Neuro-Psychiatric**	Depression/Anxiety	2	6	1	3	1	3	0	0
**Other**	Infusion reaction	2	6	0	0	2	6	0	0

* One patient with a history of gastroesophageal reflux disease and irritable bowel syndrome developed grade 3 nausea, vomiting, gastroparesis and achalasia after one dose of ipilimumab. One case of grade 4 uric acid elevation was considered possibly related to ipilimumab.

### Biomarkers

**Figure 1:**
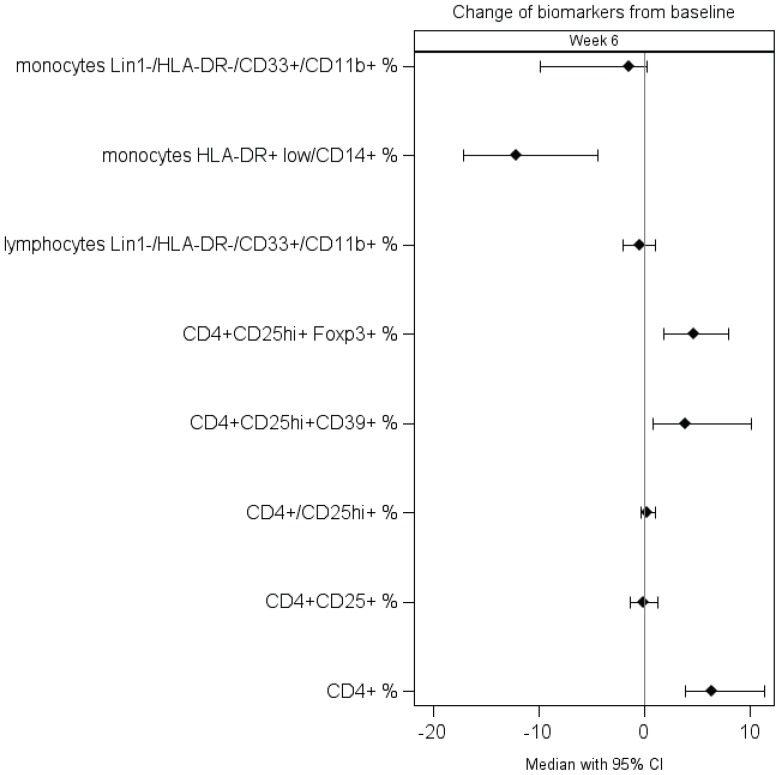
Forest plot of the multicolor flow cytometry data comparing the cell surface marker expression of regulatory T cells (Treg) and myeloid-derived suppressor cells (MDSC) on peripheral blood mononuclear cells (PBMC) at baseline and following treatment with ipilimumab (6 weeks). The plot represents average within-patient changes from baseline to 6 weeks (with corresponding 95% confidence intervals). Treg were defined as cells expressing (1) CD4^+^CD25hi^+^FOXP3^+^ or (2) CD4^+^CD25hi^+^CD39^+^ activated T cells (CD3^+^CD4^+^CD25^+^). MDSC were defined as cells expressing (1) Lin1−/HLA-DR−/CD33+/CD11b+ lymphoid gate, (2) Lin1^−^/HLA-DR^−^/CD33^+^/CD11b^+^ monocyte gate or (3) HLA-DR^+lo^/CD14^+^ monocyte gate (N = 27 patients). Examples of raw data are provided in Figures S3 (T-reg gating) and S4 (MDSC gating).

**Figure 2:**
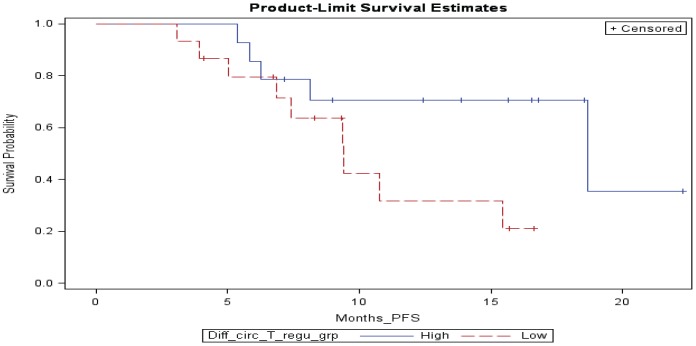
Kaplan-Meier plot of progression free survival (PFS) by the dichotomized (at median) change in the percentage of circulating regulatory T cells (Treg) between baseline and week 6. Greater increase in circulating Treg (CD4+CD25hi+Foxp3+%) was associated with improved PFS (HR = 0.57, p = 0.034; N = 27 patients). Example of raw data is provided in Figure S3 where the gating strategy used for Treg is shown.

*Peripheral cellular immune monitoring by multicolor flow cytometry* (N = 27 patients with available samples tested). Changes in circulating Treg and MDSC were compared between baseline and 6 weeks after ipilimumab. There was a significant increase in the percentage of circulating Treg (CD4+CD25hi+Foxp3+ and CD4+/CD25hi+/CD39+) as shown in Figure 1. Greater increase in circulating Treg (CD4+CD25hi+Foxp3+) was found to be associated with improved PFS (p = 0.034; HR = 0.57); Figure 2. Greater increase in CD4+/CD25hi+ T cells was also associated with improved PFS (p = 0.043; HR = 0.62).

**Figure 3:**
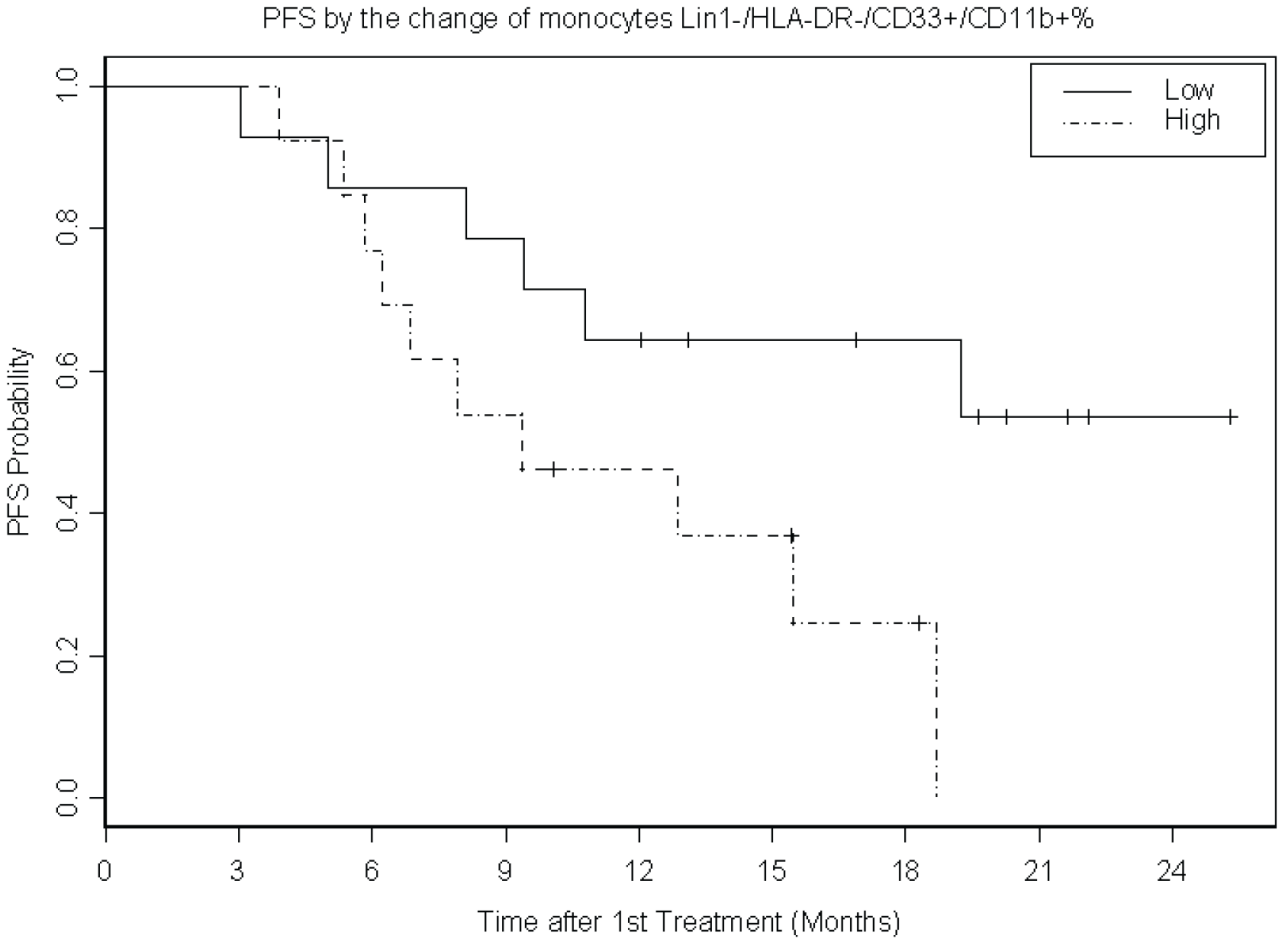
Kaplan-Meier plot of progression free survival (PFS) by the dichotomized change in the percentage of circulating MDSC between baseline and week 6. Greater decrease in circulating monocyte gate MDSC (Lin1−/HLA-DR−/CD33+/CD11b+%) was associated with improved progression free survival (PFS; p = 0.03; N = 27 patients). Example of raw data is provided in Figure S4 where the gating strategies used for MDSC subsets are shown.

In terms of MDSC, there was a decrease in the percentage of all MDSC populations tested at 6 weeks as compared to baseline, most significantly for the monocyte gate MDSC HLA-DR^+lo^/CD14^+^ (p<0.0001); Figure 1. Greater decrease in circulating monocyte gate MDSC Lin1^−^/HLA-DR^−^/CD33^+^/CD11b^+^ was associated with improved PFS (p = 0.03) as illustrated in Figure 3.

We detected evidence of spontaneous *in vivo* cross presentation resulting in type I (interferon-γ producing), fully activated (CD69^+^) CD4^+^ and CD8^+^ antigen-specific T-cell immunity against cancer-testis (NY-ESO-1) as well as melanocytic lineage (MART-1, gp100) antigens in the absence of therapeutic vaccination. These responses were significantly potentiated after ipilimumab as assessed at 6 weeks (Figure S2). We classified the changes of T cell response after ipilimumab as follows into (A) <1.5 fold over baseline, (B) 1.5–3 and (C) ≥3. Significant increases (≥3fold) in CD3^+^/CD4^+^/INF-γ^+^ T-cells were seen only in patients who were progression free at 6-months.

**Figure 4:**
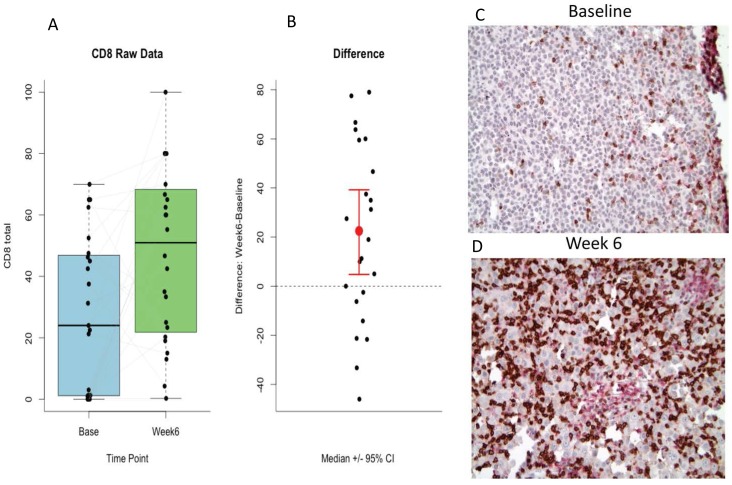
Immunohistochemistry (IHC) of CD8+ tumor infiltrating lymphocytes (TIL). There was a significant increase in CD8+ T cells from baseline to week 6 (Wilcoxon signed-rank test p = 0.02; N = 24 patients). (A) Boxplots of IHC scores of CD8+ TIL at baseline and week 6. Total counts from the same patient at the two time points are connected by light gray lines. (B) Plot of the median change in CD8+ TIL at week 6 (compared to baseline) with corresponding 95% confidence intervals. (C–D) Example of baseline and week 6 tumor CD8+ TIL (stained brown) by IHC. Magnification: 20×.

*Immunohistochemistry* (N = 24 patients with available samples tested). In tumor, there was significant increase in CD8^+^ tumor infiltrating lymphocytes observed after ipilimumab (p = 0.02) as illustrated in Figure 4. No significant change in CD20^+^ B cells was seen between baseline and 6 weeks. However, low levels of baseline and post-treatment tumor CD20^+^ B cells tended to be associated with worse clinical response (p = 0.07) and with worse PFS (p = 0.06), respectively. Among the remaining antibodies tested, no other significant findings by IHC were found.

*Tumor immune monitoring by flow cytometry* (N = 10 patients with available samples tested). Compared to baseline, there was increased tumor infiltration following ipilimumab by fully activated (CD69^+^) CD3^+^/CD4^+^ T cells (mean change = 19; SD = 14, p = 0.06) and CD3^+^/CD8^+^ T cells (mean change = 11; SD = 19; p = 0.2). There was evidence of induction/potentiation of memory T cells expressing cytokine (CD3^+^/CD8^+^/CD45RO^+^/TNF-α^+^; mean change = 1.38; SD = 1.46; p = 0.03) but not naïve (CD3^+^/CD8^+^/CD45RO^−^/TNF-α^+^; p = 0.44) T cells at 6 weeks.

There was a trend towards an inverse association between the change in Treg in tumor and clinical-benefit (CR/PR/SD versus PD; p = 0.09). CD4^+^CD25hi^+^FOXP3^+^ Tcells tended to be higher at week 6 (mean change = 1.5; SD = 1.46) in the PD group while the opposite was observed in the clinical-benefit group (mean change = −0.64, SD = 1.83). Greater decrease in tumor MDSC Lin1^−^/HLA-DR^−^/CD33^+^/CD11b^+^ was associated with improved PFS at one year (p = 0.04).

## Discussion

The neoadjuvant application of ipilimumab in this trial has allowed the monitoring of the immunomodulatory effects of ipilimumab in the circulation and the tumor microenvironment of patients treated and the testing of mechanistic hypotheses. Clinically, the evaluation of efficacy was meant to be descriptive given the small sample size and the lack of a control group. The study enrolled patients with melanoma recurrence and mortality risk that is the highest among those considered potentially operable, including 27 patients with recurrent disease. In addition, 18 patients had a component of in-transit metastatic disease. The PFS and OS rates observed may therefore be considered favorable, with the noted caveats of the statistical limitations of small single arm studies. The most common adverse events related to ipilimumab were immune mediated, were consistent with the known toxicity profile of this agent at 10 mg/kg and were manageable utilizing established toxicity management guidelines [9], [11], [28].

The outcomes of the peripheral monitoring of Treg and MDSC as mediators of immune suppression were consistent with our prior observations in patients with metastatic melanoma treated with the combination of tremelimumab and HDI [14], [29]. The significant increase in circulating Treg paralleled an increase in the overall CD4^+^ T cell population. This was expected with CTLA-4 blockade since Treg express CTLA-4 in basal conditions. More interesting was the observation that a greater increase in circulating Treg was associated with improved PFS. This raises questions about the functional status of these Treg that should be further pursued, although the opposite change in Treg was observed in the TME and the clinical activity does not appear to have been negatively affected by the circulating Treg increase. These findings are consistent with our report of a similar impact of tremelimumab/HDI on circulating Treg in metastatic melanoma in the presence of significant clinical activity, but no post treatment tumor samples were available to assess Treg in the TME in that study [29]. Moreover, it is interesting to note that Ménard et al. had demonstrated that CTLA-4 blockade with tremelimumab in advanced melanoma patients restored the circulating effector and memory CD4+ and CD8+ T cell pool and TCR-dependent T-cell proliferation that became resistant to Treg-mediated suppression [30]. In tumor, in this study, there was a trend towards an inverse association between the change in Treg after ipilimumab and clinical outcome. Hamid, et al reported a significant association between high baseline tumor FOXP3 expression by IHC and improved clinical outcome after ipilimumab treatment for metastatic melanoma supporting a role for ipilimumab in modulating intra-tumoral Treg [31]. We examined CD4^+^CD25^hi^FOXP3^hi^ Treg, the most frequently examined “nTreg” subset. In an exploratory analysis, we examined the Treg for the suppressive ectonucleotidase CD39 and on average, they were 55% positive (range 15–93% positive), further indicating suppressive nTreg [32].

In monitoring MDSC in metastatic melanoma patients treated with the combination of tremelimumab/HDI, we first reported a significant regulatory impact on these cellular mediators of immunosuppression in a trial involving CTLA-4 blockade [29]. We have now documented a similar regulatory impact of ipilimumab alone upon MDSC in the neoadjuvant setting. This effect of ipilimumab was also seen in tumor where a greater decrease in tumor MDSC was associated with improved PFS at one year. While there is substantial variation in identification of human MDSC, we examined three different subsets which have been supported by previous studies, and found that the CD14^+^/HLA-DR^lo^ subset was highly significantly changed, which is a key MDSC subset in the recent study of Walter et al [33]. Decrease in the Lin^−^/CD11b^+^/CD33^+^ monocyte subset was significantly correlated with improved PFS, which was also seen in other cancer settings [17], [19], and was a particularly suppressive subset examined in renal cell carcinoma and sarcoma patients [34]. These observations with Treg and MDSC at baseline as well as early on-treatment support a significant immunomodulatory role of anti-CTLA-4 therapy in relation to the Treg and MDSC host responses. They support further testing of these mediators of immune suppression as predictors of clinical benefit as baseline or early-on-treatment markers.

Patients were not immunized with defined tumor antigens, however we hypothesized that ipilimumab might allow the development of T cell responses to antigens expressed by melanoma or allow the expansion of spontaneous immunity. We detected evidence of *in-vivo* cross presentation of immunogenic shared melanoma tumor antigens, and stimulation of tumor antigen-specific type I, activated, CD4^+^ and CD8^+^ T cell responses at baseline that were augmented with ipilimumab therapy in the blood. Cellular responses were detected against cancer-testis (NY-ESO-1) as well as melanocytic lineage (MART-1, gp100) antigens in the absence of vaccination. This observation, at the least, does not support a therapeutic utility for exogenous tumor immunization in combination with ipilimumab in the absence of evidence of benefit to date. It is noteworthy that the pivotal phase III study of ipilimumab at 3 mg/kg with or without gp100 peptide vaccine in metastatic melanoma showed no added therapeutic impact of the peptide vaccine compared to ipilimumab monotherapy [9]. Our observation that the most significant increases (≥3 fold) in CD3^+^/CD4^+^/IFN-γ^+^ T cells was seen only in patients who remained progression-free at 6-months supports further exploring this cellular subpopulation as a potential tool for early assessment of treatment outcome. This potential therapeutic predictive tool is similar to the observation by Carthon, et al in relation to CD4^+^ICOS^hi^ T cells [35]. In bladder cancer patients treated preoperatively with ipilimumab, CD4^+^ and CD8^+^ ICOS^hi^ T cells were increased compared with baseline, and patients with clinical benefit at week 24 had persistent elevation in the percentage of CD4^+^ICOS^hi^ T cells; only 1/7patients with progressive disease or death at week 24 had persistent elevation in the percentage of CD4^+^ICOS^hi^ T cells [35].

By IHC, we observed a significant influx of CD8^+^ T cells into tumor following ipilimumab. It was interesting to note evidence of induction/potentiation of T cell memory (CD45RO^+^) but not naïve (CD45RO^−^) T cells in the tumor biopsies examined by flow cytometry. Taken together with the data of Galon, et al [36], [37] regarding the prognostic value of CD3^+^, CD8^+^ and CD45RO^+^ cells in relation to survival in colorectal cancer, our data suggest a role for ipilimumab in inducing and/or potentiating such effector elements in tumor, eventually translating into the clinical benefits seen with this agent. This is in addition to a potential therapeutic predictive role for these biomarkers that can be assessed in tumor biopsies obtained at baseline or early on-treatment.

Low baseline tumor infiltrating CD20+ B cells showed a trend towards association with worse clinical response (p = 0.07; N = 24). While not statistically significant, this trend is interesting in view of the report by DiLillo, et al that B cell depletion in mice enhanced B16 melanoma outgrowth [38]. These authors suggested that B cells are required for optimal CD4^+^ and CD8^+^ T cell tumor immunity, noting that effector-memory and IFNγ– or TNFα–secreting CD4^+^ and CD8^+^ T cell induction was significantly impaired in B cell-depleted mice with tumors. In addition, tumor Ag-specific CD8^+^ T cell proliferation was impaired in tumor-bearing mice lacking B cells. Slingluff et al, studying immune cells infiltrating the microenvironment of melanoma metastases found that B cells are correlated with increased survival [39]. These and our observations argue for further research into the role of B cells in the tumor microenvironment and the potential supportive role of B cells for optimal CD4^+^ and CD8^+^ T cell tumor immunity. They also support further investigations into the therapeutic predictive value of in-tumor B cells, possibly as part of a predictive immune signature.

## Conclusions

This neoadjuvant approach to the evaluation of ipilimumab in high-risk locally/regionally advanced melanoma has revealed baseline and early on-treatment biomarkers of immunomodulation that provide insight to the mechanism of ipilimumab, and warrant further pursuit in future studies aiming to individualize and optimize the immunotherapy of melanoma.

## Supporting Information

Figure S1 **Kaplan – Meier plot of the probability of progression-free survival (N = 33). The estimated median is 10.8 months (95% Confidence Interval = 6.2, 19.2).**
(TIF)

Figure S2 **Forest Plot of Type I CD4 and CD8 Antigen-specific T Cell Immunity (N = 27).** T cell immunity to shared melanoma antigens was measured with peptide pools, as described in the methods. Activated (CD69+) and IFNγ-producing T cells were measured.
(TIF)

Figure S3 **The gating strategy used for regulatory T cells (Treg) is shown. Lymphocytes were gated on, and the CD4^+^CD25^hi+^ were gated on, and then histograms show the % intracellular FOXP3 and % surface CD39^+^ on those CD4^+^CD25^hi+^ lymphocytes.** A representative patient sample is shown.
(TIF)

Figure S4 **The gating strategies used for MDSC subsets are shown.** The lymphocyte, monocyte and granulocyte populations are shown by FSC and SSC. The lineage negative (lin^−^) HLA-DR^neg^ cells were then gated on, and the percent of CD11b^+^/CD33^+^ cells were enumerated. Alternatively, the CD14^+^ monocytes were gated on and the HLA-DR^lo+^ cells were identified by histogram (bottom left). A representative patient sample is shown.
(TIF)

Figure S5 **The gating strategy for identification of melanoma tumor antigen-specific CD8^+^ and CD4^+^ T cells is shown.** Lymphocytes were gated on, and the CD3^+^/CD8^+^ (top) or CD3^+^/CD4^+^ (bottom) were gated on. These cells were then assayed for CD69 and intracellular IFNγ. Shown are negative controls, PMA/ionomycin-stimulated positive control and responses NY-ESO-1 peptides. A representative patient sample is shown.
(TIF)

Table S1 **Summarizes ipilimumab administration presented by the cycle of ipilimumab and the corresponding number of patients treated.**
(DOC)

Table S2 **Six patients who developed disease progression during follow up on study achieved objective response or no evidence of disease (NED) status with subsequent therapy.**
(TIF)
