# Supplementary figures and images for: Immune Monitoring of the Circulation and the Tumor Microenvironment in Patients with Regionally Advanced Melanoma Receiving Neoadjuvant Ipilimumab
Source: PLoS One. 2014 Feb 3;9(2):e87705. doi: 10.1371/journal.pone.0087705 (PMC3912016; doi:10.1371/journal.pone.0087705)

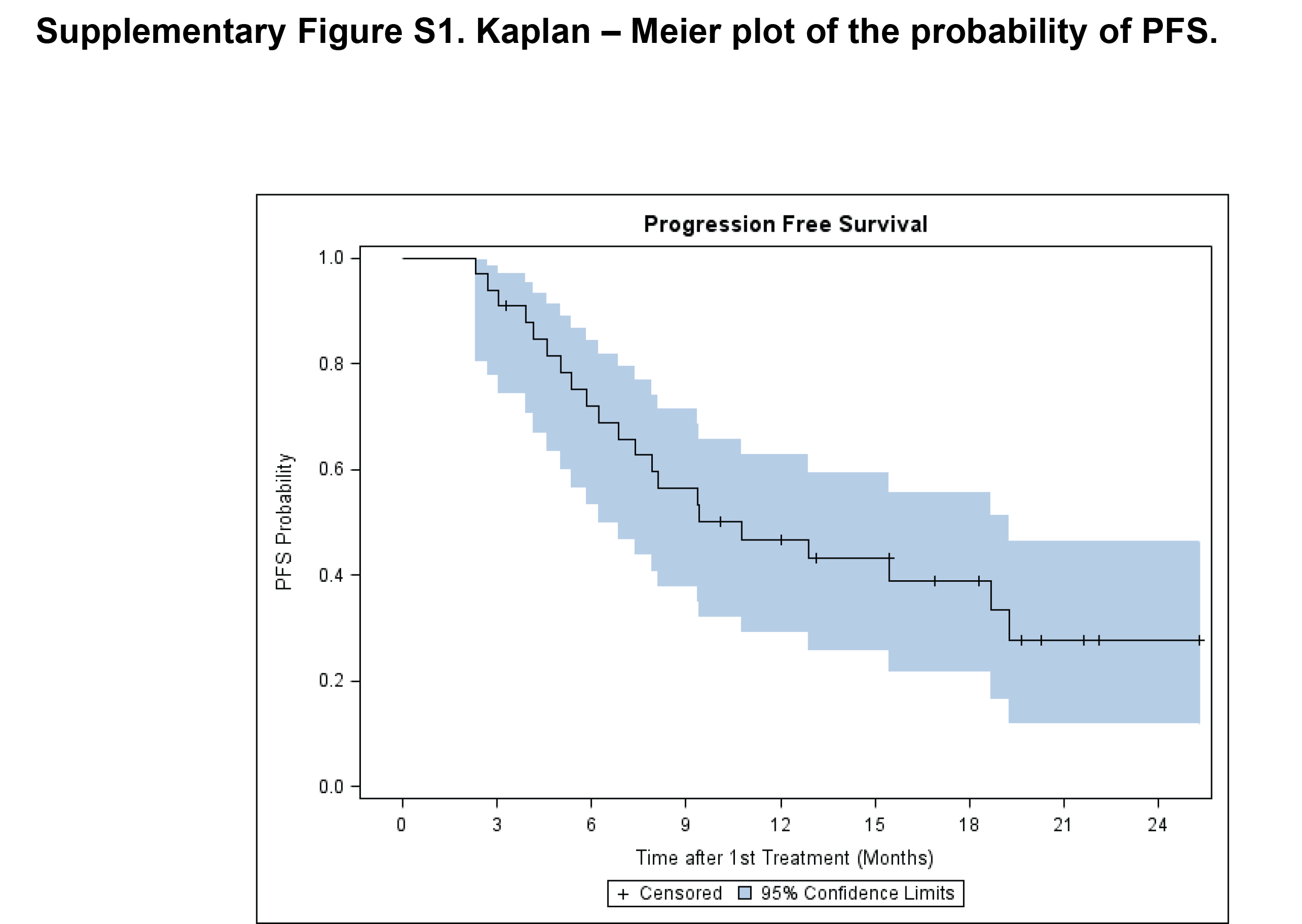

Supplement: Figure S1 — Kaplan – Meier plot of the probability of progression-free survival (N = 33). The estimated median is 10.8 months (95% Confidence Interval = 6.2, 19.2). (TIF) [file pone.0087705.s001.tif]

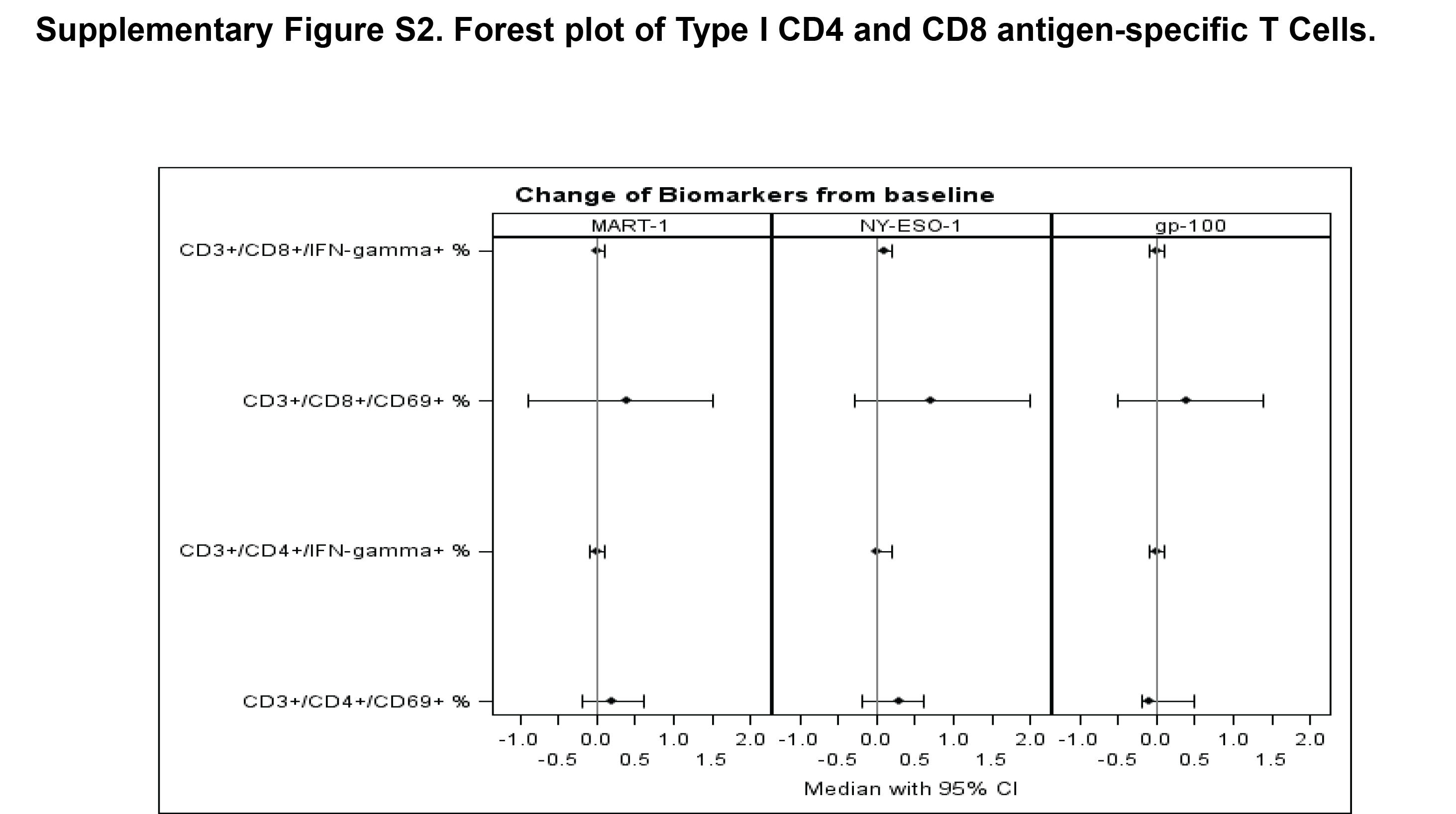

Supplement: Figure S2 — Forest Plot of Type I CD4 and CD8 Antigen-specific T Cell Immunity (N = 27). T cell immunity to shared melanoma antigens was measured with peptide pools, as described in the methods. Activated (CD69+) and IFNγ-producing T cells were measured. (TIF) [file pone.0087705.s002.tif]

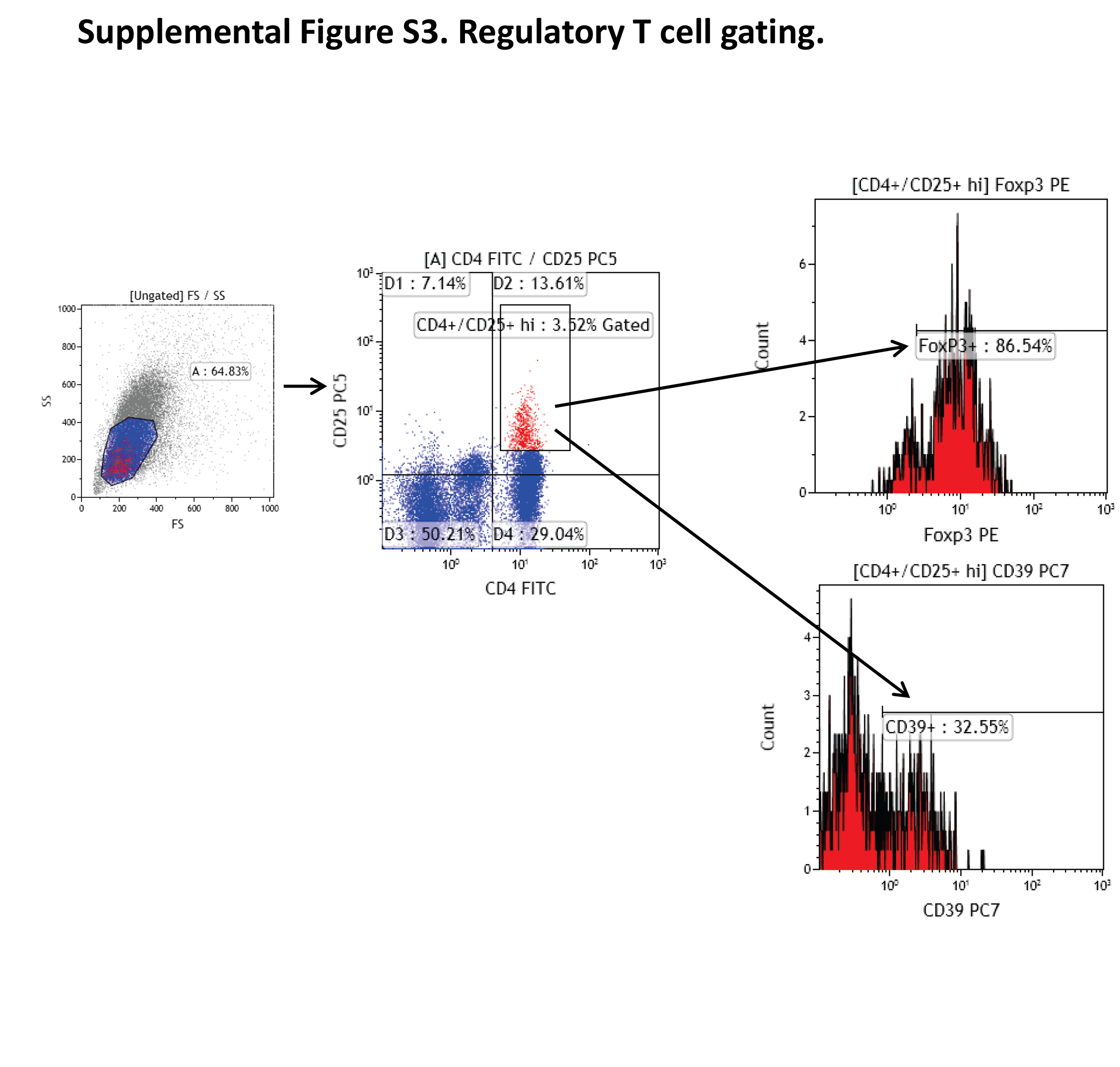

Supplement: Figure S3 — The gating strategy used for regulatory T cells (Treg) is shown. Lymphocytes were gated on, and the CD4+CD25hi+ were gated on, and then histograms show the % intracellular FOXP3 and % surface CD39+ on those CD4+CD25hi+ lymphocytes. A representative patient sample is shown. (TIF) [file pone.0087705.s003.tif]

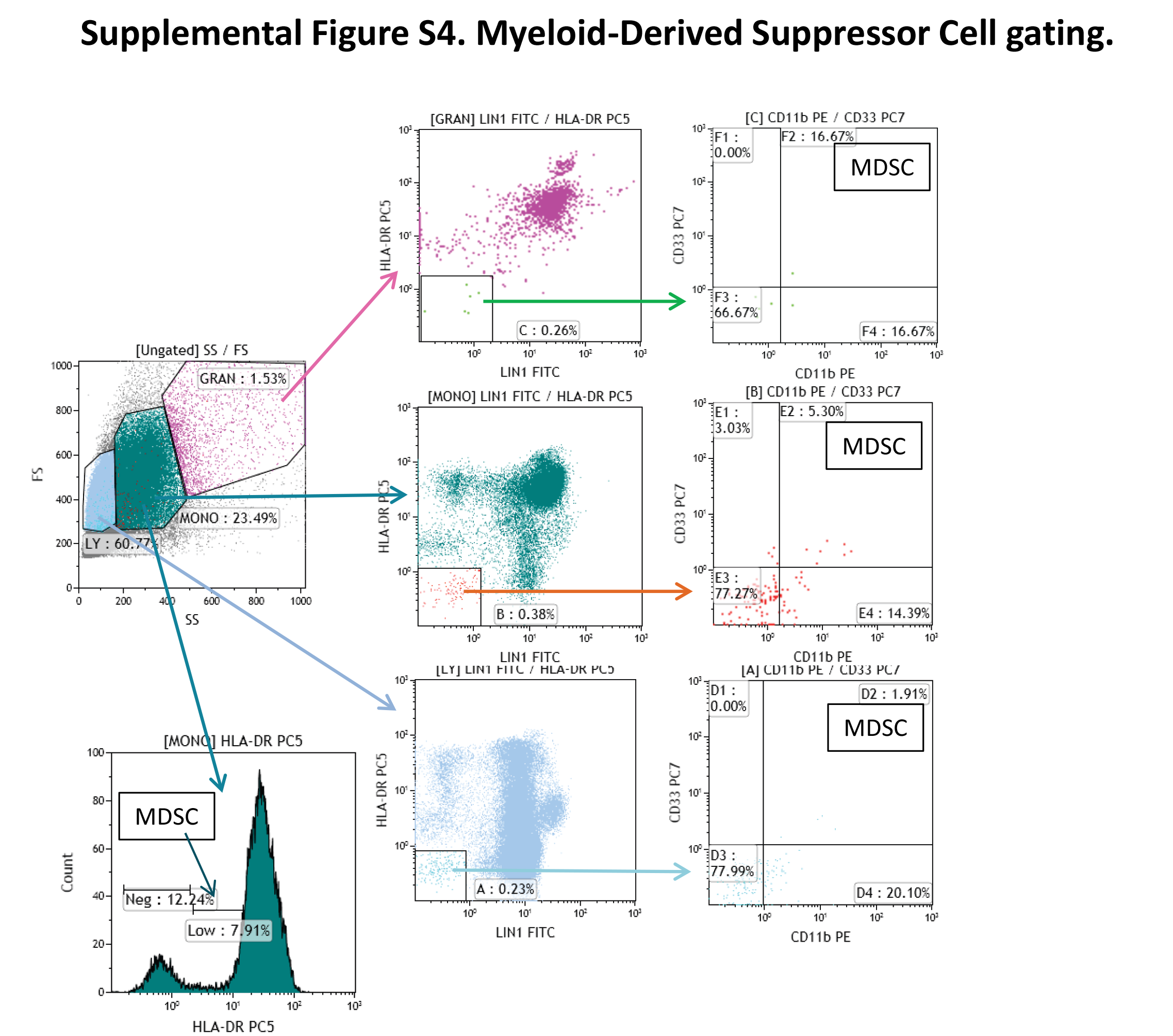

Supplement: Figure S4 — The gating strategies used for MDSC subsets are shown. The lymphocyte, monocyte and granulocyte populations are shown by FSC and SSC. The lineage negative (lin−) HLA-DRneg cells were then gated on, and the percent of CD11b+/CD33+ cells were enumerated. Alternatively, the CD14+ monocytes were gated on and the HLA-DRlo+ cells were identified by histogram (bottom left). A representative patient sample is shown. (TIF) [file pone.0087705.s004.tif]

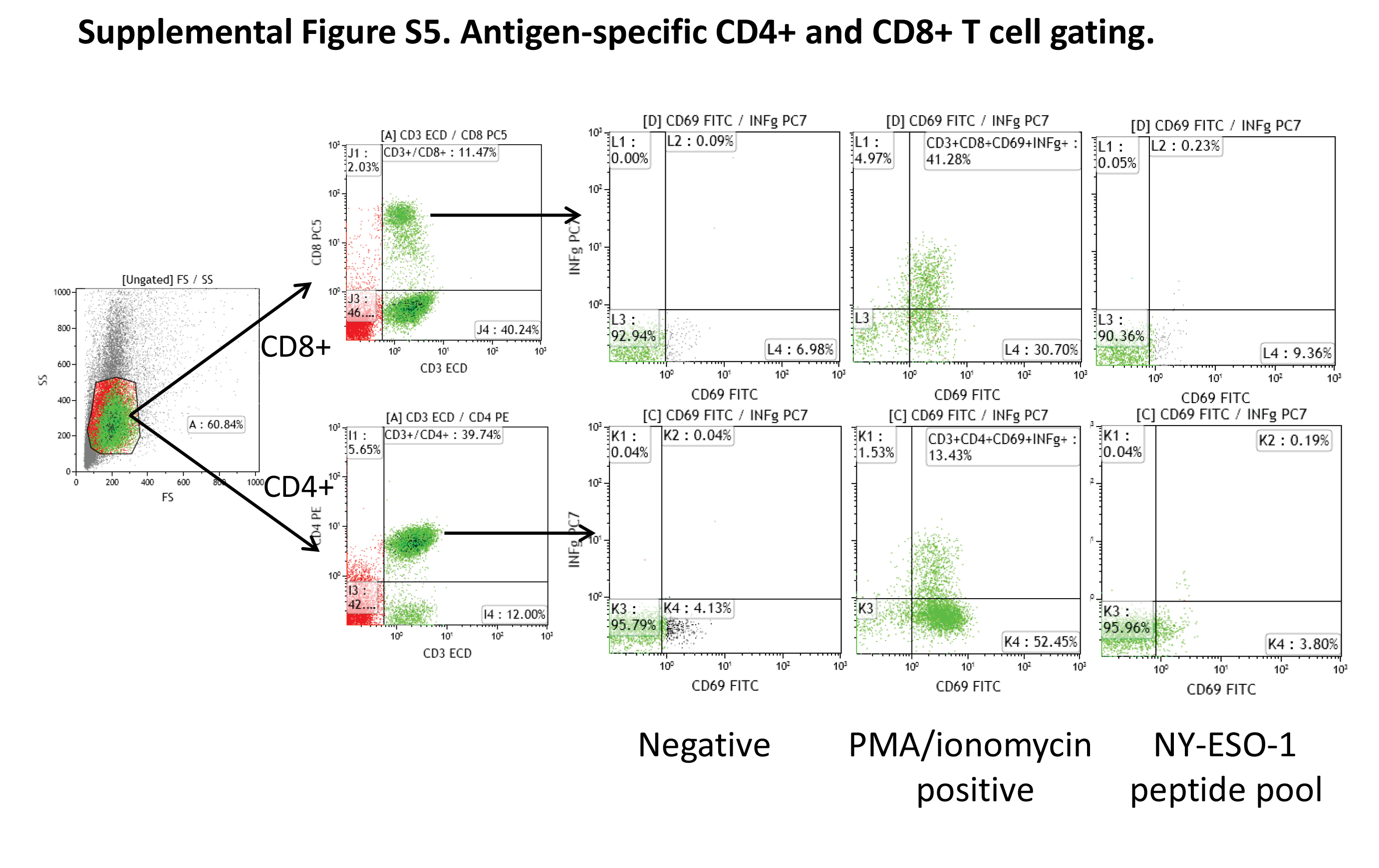

Supplement: Figure S5 — The gating strategy for identification of melanoma tumor antigen-specific CD8+ and CD4+ T cells is shown. Lymphocytes were gated on, and the CD3+/CD8+ (top) or CD3+/CD4+ (bottom) were gated on. These cells were then assayed for CD69 and intracellular IFNγ. Shown are negative controls, PMA/ionomycin-stimulated positive control and responses NY-ESO-1 peptides. A representative patient sample is shown. (TIF) [file pone.0087705.s005.tif]

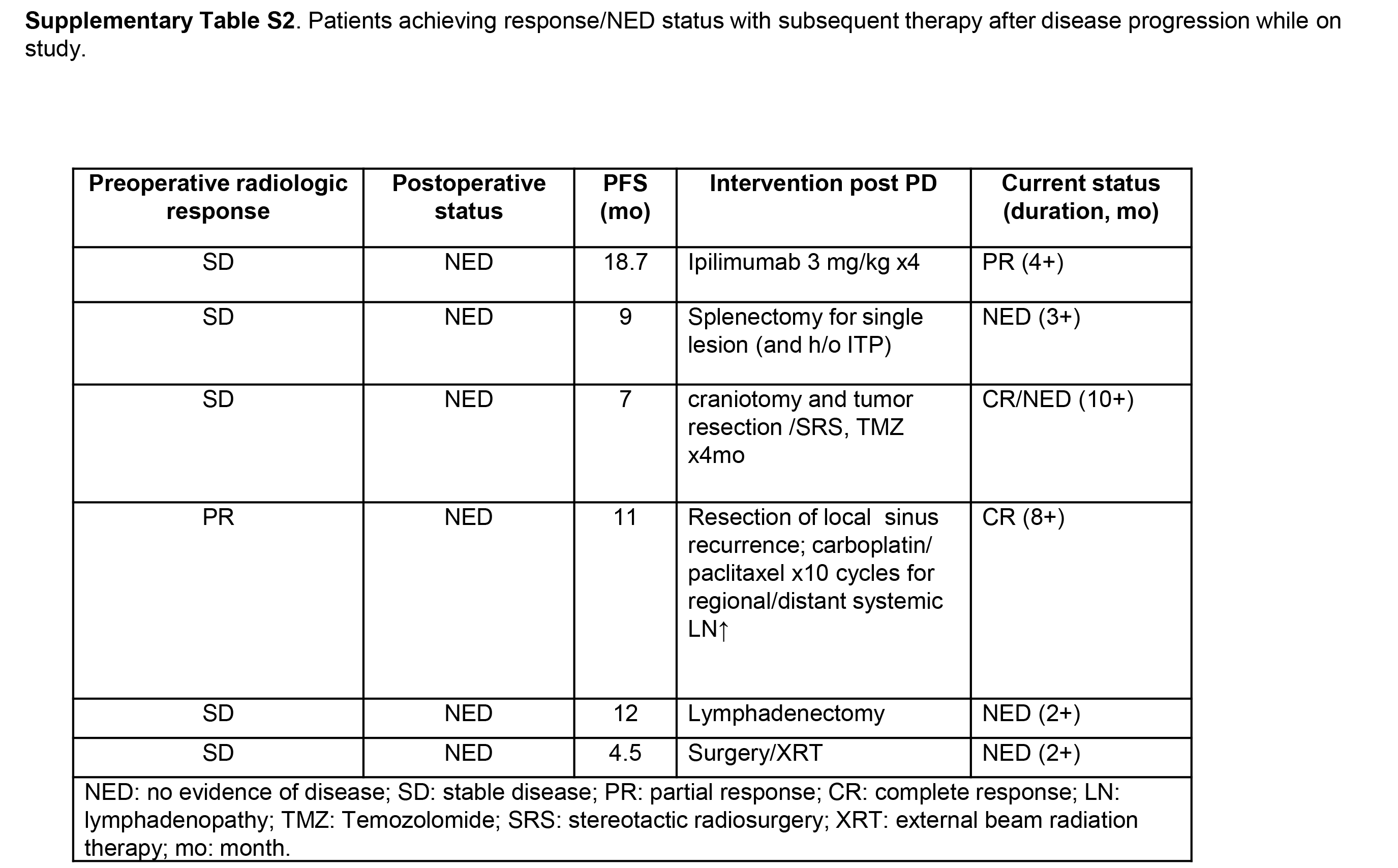

Supplement: Table S2 — Six patients who developed disease progression during follow up on study achieved objective response or no evidence of disease (NED) status with subsequent therapy. (TIF) [file pone.0087705.s007.tif]
