# Supplementary material for: Immune Monitoring of the Circulation and the Tumor Microenvironment in Patients with Regionally Advanced Melanoma Receiving Neoadjuvant Ipilimumab
Source: PLoS One. 2014 Feb 3;9(2):e87705. doi: 10.1371/journal.pone.0087705 (PMC3912016; doi:10.1371/journal.pone.0087705)
Supplement: Table S1 — Summarizes ipilimumab administration presented by the cycle of ipilimumab and the corresponding number of patients treated. (DOC) [file pone.0087705.s006.doc]

| **Supplementary Table S1**. Ipilimumab administration presented by the cycle of ipilimumab and the number of patients treated | | | | |
| --- | --- | --- | --- | --- |
| **Cycle** | **No. patients treated (%)** | **No. patients off treatment after this cycle** | **No. with PD as Reason for D/C** | **No. with Toxicity as Reason for D/C** |
| **1** | 35/35 (100) | 3 | 1* | 2 |
| **2** | 32/35 (91) | 11 | 4* | 7 |
| **3** | 21/35 (60) | 3 | 1 | 2^‡^ |
| **4** | 18/35 (51) |  |  |  |
| *One patient with stage IV disease, considered non-evaluable for efficacy.  ^‡^One patient declined protocol-allowed cycle 4 of ipilimumab after resolution of grade 2 diarrhea.  PD: disease progression; D/C: discontinue treatment. | | | | |
